# Comparison of Clinical and Radiographic Success Rates of Pulpotomy in Primary Molars using Ferric Sulfate and Bioactive Tricalcium Silicate Cement: An *in vivo* Study

**DOI:** 10.5005/jp-journals-10005-1425

**Published:** 2017-06-01

**Authors:** Kavita Sirohi, Mohita Marwaha, Anil Gupta, Kalpana Bansal, Ankit Srivastava

**Affiliations:** 1 Postgraduate Student, Department of Pedodontics and Preventive Dentistry SGT Dental College, Hospital & Research Institute, Gurugram Haryana, India; 2 Reader, Department of Pedodontics and Preventive Dentistry SGT Dental College, Hospital & Research Institute, Gurugram Haryana, India; 3 Professor and Head, Department of Pedodontics and Preventive Dentistry SGT Dental College, Hospital & Research Institute, Gurugram Haryana, India; 4 Assistant Professor, Department of Pedodontics and Preventive Dentistry, Centre for Dental Education and Research, AIIMS, New Delhi, India; 5 Reader, Department of Pedodontics and Preventive Dentistry SGT Dental College, Hospital & Research Institute, Gurugram Haryana, India

**Keywords:** Biodentine, Ferric sulfate, Primary molars, Pulpotomy.

## Abstract

**Introduction:**

Formocresol has been a popular pulpotomy medicament for many years. It is considered the “gold standard“ in pediatric dentistry. However, concerns have been raised over its use in children. It has been reported that formocresol has toxic and mutagenic effects in cell culture, dental crypts, and precancerous epithelial cells. Therefore, additional biocompatible treatment alternatives are required to replace formocresol pulpotomy.

**Aims:**

This study compared the clinical and radiographic success rates of ferric sulfate (FS) and bioactive tricalcium silicate cement (Biodentine, Septodont) as pulpotomy agents in primary molar teeth over a period of 9 months.

**Materials and methods:**

Fifty primary molar teeth, symptom free, requiring pulpotomy in children aged 4 to 8 years were treated with conventional pulpotomy procedures. Ferric sulfate 15.5% solution (applied for 15 second for 25 teeth) and Biodentine (for 25 teeth) were used as pulpotomy agents. Permanent restorations were stainless steel crowns in most cases, in both groups. Patients were recalled for follow-up at 1, 3, 6, and 9 months intervals. The data were statistically analysed using chi-square test.

**Results:**

At 9 months, 96% clinical success rate was observed in the FS and 100% in the Biodentine group. Radiographic success rate in the FS group was 84%, whereas 92% in the Biodentine group at 9 months. No statistically significant difference was found between the two groups.

**Conclusion:**

Biodentine can be used as a pulpotomy agent but further long-term studies are required.

**How to cite this article:**

Sirohi K, Marwaha M, Gupta A, Bansal K, Srivastava A. Comparison of Clinical and Radiographic Success Rates of Pulpotomy in Primary Molars using Ferric Sulfate and Bioactive Tricalcium Silicate Cement: An *in vivo* Study. Int J Clin Pediatr Dent 2017;10(2):147-151.

## INTRODUCTION

The primary objective of pulp therapy is to maintain the integrity and health of the teeth and their supporting structures. The main aim is to maintain the vitality of the pulp of a tooth affected by caries, traumatic injuries, or any other causes.^1^ Furthermore, retaining primary teeth will also maintain function (mastication, phonation and swallowing) and esthetics. Pulpotomy is the ablation of infected or affected pulp tissues, leaving the residual vital pulp tissues intact, thus preserving vitality and function (totally or partially) of the radicular pulp, and the remaining pulp stump is covered with a capping agent.^2^ It is indicated when caries removal results in pulp exposure in a primary tooth with a normal pulp or reversible pulpitis or after a traumatic pulp exposure.^1^

Formocresol has been a popular pulpotomy medicament for many years. It is considered as “gold standard“ in pediatric dentistry. However, concerns have been raised over its use in children. It has been reported that formocresol has toxic and mutagenic effects in cell culture, dental crypts, and precancerous epithelial cells. Therefore, additional biocompatible treatment alternatives are required to replace formocresol pulpotomy.^3^ A wide range of materials other than formocresol, such as glutaraldehyde, ferric sulfate (FS), electrosurgery, laser, collagen, freeze dried bone, morphogenic bone proteins, and mineral trioxide aggregate (MTA) have been used for pulpotomy.^2^

Ferric sulfate has also been used as a pulpotomy agent because of its hemostatic properties. It forms a metal protein clot at the surface of the pulp stump and this act as a barrier to irritating components of the subbase.^4^ Even though, high clinical success rates have been found by using FS, studies have shown that it might produce moderate to severe inflammatory responses histologically.^5^ Other regeneration techniques with preservation methods are also recommended.^6^

Calcium silicate-based materials (CSMs), such as MTA have been largely used as endodontic repair materials because of their superior seal, biocompatibility, and regenerative capabilities. One such recently introduced bioactive CSMs is Biodentine (Septodont, St. Maur-des-Fosses, France).^6^ It shares both its indications and mode of action with calcium hydroxide, but does not have its disadvantages. Biodentine™ consists of powder in a capsule and liquid in a pipette.^7^ It contains tricalcium silicate, dicalcium silicate, calcium carbonate, iron oxide, and zirconium oxide as its powder components and calcium chloride and a water soluble polymer as its liquid components.^6^ It can be used on both crown and root.^7^ It is recommended for use as pulp protection, direct and indirect pulp-capping, pulpotomy, dentin substitute in sandwich restorations, endodontic repair, apexification, and retrograde root canal obturation. This material exhibits the same excellent biological properties as MTA and can be placed in direct contact with dental pulp.

Thus, the purpose of this study was to compare the clinical and radiographic success rate of pulpotomy in primary molars using FS and bioactive tricalcium silicate (Biodentine) cement over a period of 9 months.

## MATERIALS AND METHODS

Fifty primary molars requiring pulpotomy in 4 to 8 years-old children free from any systemic disease, who reported to the Department of Pedodontics and Preventive Dentistry, SGT Dental College, Hospital and Research Institute, Gurugram, Haryana, India, were selected. The procedure, possible discomfort, and benefits were explained fully to the parents of the children involved prior to the treatment. Ethical clearance was obtained from the ethical committee of the institute. Verbal and written consent was obtained from the parents of the subjects enrolled in the study. Selection criteria for teeth included in the study were as follows: (1) Primary molars with vital carious pulp exposure, (2) symptomless teeth with deep carious lesions, (3) no clinical or radiographic evidence of pulp degeneration (swelling or sinus tract, furcation radiolucency, internal and external resorption, periapical radiolucency, pathological mobility, history of spontaneous, and nocturnal pain), (4) teeth should be restorable, and (5) at least 2/3rd of the root length was present.^8^

All the teeth were assigned randomly to one of the two treatment groups: FS (group I) and Biodentine (group II). A total of 25 primary molars were present in each of the two groups.

The conventional pulpotomy procedure was carried out step by step in one visit. Local anesthesia (2% xylo-caine) was administered and all the teeth were isolated with rubber dam. After caries removal, coronal access was obtained using a (No. 330) high speed bur mounted in an airotor with water spray to expose pulp chamber. Deroofing of pulp chamber was done. A sterile sharp spoon excavator was used to excise the coronal pulp. After the coronal pulp was amputated, pulp chamber was irrigated with normal saline to wash away dentin debris. Cotton pellets wet with sterile saline were applied for few minutes on amputated pulp stumps with pressure to achieve hemostasis.

After the standardized technique, the teeth assigned for group I were treated with 15.5% FS solution (Astringe-dent, Ultradent products, USA). The FS was placed over amputated pulp stump with the help of an applicator tip for 15 seconds and then the pulp chamber was flushed with water by an air-water syringe, dried by cotton pellet. If hemostasis was achieved, a thick mix of zinc oxide eugenol (ZOE) paste was placed over the pulp stumps. IRM base (dentsply) was given and the teeth were restored with composite restorative material (coltene) in the same visit. If haemorrhage persisted, pulpectomy was done and the tooth was eliminated from the study.

In group II, pulp stumps were covered with bioactive tricalcium silicate cement (Biodentine), prepared according to manufacturer’s instructions. Five drops of liquid was added to the powder supplied in the capsule and mixed in the amalgamator for 30 seconds. The mixture was delivered to the pulp stumps with the help of plastic filling instrument or the plastic spatula provided by the manufacturer, and condensed with moistened sterile cotton pellet. The glass ionomer cement (GIC) base was given over the bioactive tricalcium silicate cement. Teeth were restored in the same visit with composite restorative material. Prefabricated stainless steel crowns (3M ESPE) were given in most of the teeth in both groups.

Pre- and postoperative radiograph were taken. The children were recalled for clinical and radiographic follow-up at 1, 3, 6, and 9 months. The success of the procedure was assessed based on clinical signs^8^ (pain, tenderness to percussion, abscess, swelling, fistula, and pathologic mobility) and radiographic findings^8^ (radicu-lar radiolucency, internal and external root resorption, periodontal ligament (PDL) space widening, and furcation radiolucency).

The available data was subjected to statistical analysis. The chi-square test was used to compare the differences between the groups. All the statistical analyses were performed with the statistical package for the social sciences version 15.0 software.

## RESULTS

Pulpotomies were performed in a total of 50 primary molars in children aged 4 to 8 years-old. Ferric sulfate (Astringedent, Ultradent, 25 teeth) and bioactive tricalcium cement (Biodentine, 25 teeth) were used as medicaments for pulpotomy. The teeth were assessed for the presence of clinical signs (pain, tenderness to percussion, abscess, swelling, fistula, and pathologic mobility) and radiographic findings (radicular radiolucency, internal and external root resorption, PDL space widening and furcation radiolucency) at 1, 3, 6, and 9 months interval. The presence of one or more findings were considered as failure.

Table 1 shows that clinical success rate for FS group was 96 and 100% for Biodentine group over a period of 9 months. In the FS group, out of 25 teeth, 1 tooth was extracted due to pain at 9 months.

Table 2 depicts that radiographic success rate for FS group was 84 and 92% for Biodentine group over a period of 9 months. Out of 25 teeth, 4 teeth in FS group and 2 teeth in Biodentine group were considered as radiographic failure. Internal resorption was the most common radiographic finding (4 teeth in FS group and 2 teeth in Biodentine group).

Table 3 shows overall success rate at 1, 3, and 6 months follow-up period: 100% overall success rate was seen in FS group. In Biodentine group, 2 out of 25 teeth were considered as failure at 6 months.

At 9 months, 5 out of 25 teeth showed failure in FS group and the overall success rate was 80%. In Bioden-tine group, 2 out of 25 teeth were considered as failure at 9 months, the overall success rate for Biodentine group was 92%. Although, statistically significant difference was not seen between the two groups, Biodentine group showed a higher overall success rate (92%) at 9 months when compared with that of FS (80%).

## DISCUSSION

The preservation of the primary dentition for as long as possible is of great importance so as to maintain arch length and preserve masticatory function.^9^ Endodontic procedures, such as pulpotomy and root canal treatment aim to avoid early extraction of heavily decayed teeth, and subsequently allow for a smoother transition from primary to permanent dentition.^2^

**Table 1:** **Table 1:** Clinical success rate in the FS and Biodentine group at 1, 3, 6, and 9 months interval

				*1 Month*				*3 Months*				*6 Months*				*9 Months*			
				*Absent*		*Present*		*Absent*		*Present*		*Absent*		*Present*		*Absent*		*Present*	
Pain		Ferric sulfate		25		0		25		0		25		0		24		1	
		Biodentine		25		0		25		0		25		0		25		0	
TOP		Ferric sulfate		25		0		25		0		25		0		25		0	
		Biodentine		25		0		25		0		25		0		25		0	
Abscess		Ferric sulfate		25		0		25		0		25		0		25		0	
		Biodentine		25		0		25		0		25		0		25		0	
Swelling		Ferric sulfate		25		0		25		0		25		0		25		0	
		Biodentine		25		0		25		0		25		0		25		0	
Fistula		Ferric sulfate		25		0		25		0		25		0		25		0	
		Biodentine		25		0		25		0		25		0		25		0	
Mobility		Ferric sulfate		25		0		25		0		25		0		25		0	
		Biodentine		25		0		25		0		25		0		25		0	

**Table 2:** **Table 2:** Radiographic success rate in the FS and Biodentine group at 1, 3, 6, and 9 month interval

				*1 Month*				*3 Months*				*6 Months*				*9 Months*			
				*Absent*		*Present*		*Absent*		*Present*		*Absent*		*Present*		*Absent*		*Present*	
Radicular radiolucency		Ferric sulphate		25		0		25		0		25		0		23		2	
		Biodentin		25		0		25		0		24		1		24		1	
Internal/externalresorption		Ferric sulphate		25		0		25		0		25		0		21		4	
		Biodentin		25		0		25		0		23		2		23		2	
PDL widening		Ferric sulphate		25		0		25		0		25		0		24		1	
		Biodentine		25		0		25		0		25		0		25		0	
Furcation radiolucency		Ferric sulfate		25		0		25		0		25		0		23		2	
		Biodentine		25		0		25		0		24		1		24		1	

**Table 3:** **Table 3:** Overall success rate (clinical and radiographic) in the FS and Biodentine group at 1, 3, 6, and 9 month interval

				*1 Month*				*3 Months*				*6 Months*				*9 Months*			
				*Absent*		*Present*		*Absent*		*Present*		*Absent*		*Present*		*Absent*		*Present*	
Overall success		Ferric sulfate		0		25		0		25		0		25		5		20	
		Biodentine		0		25		0		25		2		23		2		23	
		Chi-square		–				–				2.083				1.495			
		p-value		–				–				0.149				0.221			

When the carious process exposes the pulp, it reacts via inflammation limited to the area close to the carious lesion. If the pulp in the root canal seems to be unaffected, pulpotomy is the treatment of choice.^10,11^

This study examined the clinical and radiographic success rate of pulpotomies with FS and Biodentine as pulpotomy medicaments in primary molars. The FS is a nonaldehyde, hemostatic compound, which forms a metal-protein complex at the surface of the pulp stump, and this act as a barrier to irritating components of the subbase.^4,12^ Heilig et al^13^ suggested that hemostasis of the remaining pulpal tissue leads to increased success of pulpotomy procedure.

In this study, 80% overall success rate was observed in the FS group, whereas Fei et al,^14^ Fuks et al,^15^ and Ibricevik et al^16,17^ reported 96% success rate with FS. However, the results of Markovic et al^18^ and Sonmez et al^19^ go in accordance with this study.

In our study, the clinical success rate for FS group was found to be 96% and radiographic success rate was 84%. Similar findings were reported by Gisoure;^20^ Ng and Messer suggested that as FS is not an antimicrobial agent and cannot heal pulp or stimulate pulp regeneration, the clinical success of FS appears to be dependent on pulp status.^21^

At 3 and 6 months follow-up, 100% clinical success was observed for FS group in this study. Similar results were reported in studies conducted by Neamatollahi et al^3^ and Erdem et al^22^ Burnett and Walker^23^ reported a radiographic success rate of 70 to 76% for the FS group which was lower than the radiographic success rate (84%) observed in this study.

Internal resorption was the most common radio-graphic finding in our study, this is in accordance with Odabas. et al. The reason cited for the same could be the thinness of primary molar roots.^24^ In this study, 4 out of 25 teeth showed internal resorption, 2 teeth showed radicular radiolucency and furcation radiolucency was also found in 2 teeth. Markovik et al^18^ observed PDL widening in 15% of teeth, whereas in our study 4% of the showed PDL widening.

In most of the published studies, as well as in this study, ZOE was used as subbase, expecting that FS may act as a barrier to the irritant components of the subbase and may function in a passive manner.^17,25^ Research has shown that ZOE in direct contact with vital pulp tissues causes moderate to severe inflammatory responses that result in chronic inflammation and necrosis. Internal resorption is a common response of pulp to chronic inflammation.^17,26^ Thus, the combination of FS with different base materials is certainly worthy of further investigation.^14^ Failures in primary teeth can also be attributed to misdiagnosis of inflammation in the radicular pulp.^3^ Even though high clinical success rates have been found using FS, studies conducted by Fuks et al and Salako et al have shown FS to produce severe inflammatory responses.^15,27^ Hence, newer materials are needed to be studied.

One recently introduced bioactive CSMs is Biodentine (Septodont, St. Maur-des-Fosses, France).^6^ Biodentine consists of powder in a capsule and liquid in a pipette. The powder is mixed with the liquid in a capsule in the triturator for 30 seconds. Once mixed, it sets in approximately 12 minutes. Calcium hydroxide is formed during the setting of the cement.^7^ In this study, Biodentine was used as a pulpotomy medicament as no studies have been conducted previously using this material. Due to the lack of literature, the results of this study cannot be compared with other studies. The results of this study showed an overall (clinical and radiographic) success rate of 92% over a 9 months follow-up period. About 100% clinical success rate was observed. Internal resorption was seen in two teeth. Although, there was no statistically significant difference between FS and Biodentine group, a higher success rate was observed in the Biodentine group.

Various *in vitro* and animal studies have documented the biocompatibility and bioactivity of Biodentine and its ability to induce pulp repair. Laurent et al^28^ showed in human, entire tooth culture model that Biodentine and ProRoot MTA induced reparative dentin synthesis after direct pulp capping due to significantly increased TGF-(31 secretion level. Zanini et al^29^ reported the ability of Bio-dentine to induce cell proliferation and biomineralization was also demonstrated *in vitro* on immortalized murine pulp cells. Tran et al^30^ suggested that reparative dentin synthesis by application of Biodentine to mechanically exposed rat pulps was further proved to be induced by the expression of osteopontin.

Biodentine has been shown to have good marginal adaptation and strength to be used as a temporary restorative material for up to 6 months in a study conducted by Koubi et al.^31^ This could be an advantage while treating children in the clinical setting as it shortens the procedure time, eliminating the need to place a separate restoration.

Thus, the results of this study showed that both FS and Biodentine can be used as pulpotomy medicaments. Although, there was no significant difference between the two groups, however, Biodentine showed a high overall success rate when compared with FS. Despite the high success rate observed in Biodentine, its high cost can be a major drawback.

## CONCLUSION

The FS and Biodentine can be successfully used as medicaments for pulpotomy. Considering the physical and biological properties of Biodentine, it can be used as a substitute for other materials as a pulpotomy agent but further long-term studies are required to judge the success of the therapy.
